# Introduction of the gross motor function classification system in Venezuela - a model for knowledge dissemination

**DOI:** 10.1186/s12887-015-0433-5

**Published:** 2015-09-04

**Authors:** Kristina Löwing, Ynes C. Arredondo, Marika Tedroff, Kristina Tedroff

**Affiliations:** Department of Women’s and Children’s Health, Karolinska Institutet, Karolinska University Hospital, Q2:07, SE-171 76, Stockholm, Sweden; Child Rehabilitation Center Mundo de Sonrisas Alta Vista, Mundo De Sonrisas Building, Puerto Ordaz, 8050 Venezuela

## Abstract

**Background:**

A current worldwide common goal is to optimize the health and well-being of children with cerebral palsy (CP). In order to reach that goal, for this heterogeneous group, a common language and classification systems are required to predict development and offer evidence based interventions. In most countries in Africa, South America, Asia and Eastern Europe the classification systems for CP are unfamiliar and rarely used. Education and implementation are required. The specific aims of this study were to examine a model in order to introduce the Gross Motor Function Classification System (GMFCS-E&R) in Venezuela, and to examine the validity and the reliability.

**Methods:**

Children with CP, registered at a National child rehabilitation centre in Venezuela, were invited to participate. The Spanish version of GMFCS-E&R was used. The Wilson mobility scale was translated and used to examine the concurrent validity. A structured questionnaire, comprising aspects of mobility and gross motor function, was constructed. In addition, each child was filmed. A paediatrician in Venezuela received supervised self-education in GMFCS-E&R and the Wilson mobility scale. A Swedish student was educated in GMFCS-E&R and the Wilson mobility scale prior to visiting Venezuela. In Venezuela, all children were classified and scored by the paediatrician and student independently. An experienced paediatric physiotherapist (PT) in Sweden made independent GMFCS-E&R classifications and Wilson mobility scale scorings, accomplished through merging data from the structured questionnaire with observations of the films. Descriptive statistics were used and reliability was presented with weighted Kappa (Kw). Spearman’s correlation coefficient was calculated to explore the concurrent validity between GMFCS-E&R and Wilson mobility scale.

**Results:**

Eighty-eight children (56 boys), mean age 10 years (3–18), with CP participated. The inter-rater reliability of GMFCS-E&R between; the paediatrician and the PT was Kw = 0.85 (95 % CI: 0.75-0.88), the PT and student was Kw = 0.91 (95 % CI: 0.86-0.95) and the paediatrician and student was Kw = 0.85 (95 % CI: 0.79-0.90). The correlations between GMFCS-E&R and Wilson mobility scale were high r_s_ =0.94-0.95 (*p* < 0.001).

**Conclusions:**

In a setting with no previous knowledge of GMFCS-E&R, the model with education, supervised self-education and practice was efficient and resulted in very good reliability and validity.

## Background

Cerebral palsy (CP) is the most common cause of major physical disability in children; the prevalence varies between 2–3 per 1000 born children [1, 2]. It is a lifelong disability which requires evidence-based multi-professional interventions, adapted to the needs of the individual [3, 4]. Children with CP represent a heterogeneous group with large variations in brain pathology, everyday functioning, and need of health care [4, 5].

An increased awareness of the vast global inequalities and accessibility to evidence-based care, as well as adapted services and adapted equipment for children with developmental disabilities and CP, has become apparent [6]. In a recent review from Africa, identification of obstacles for optimal health care was specified. The factors found included; absence of knowledge, limited access to healthcare facilities and specialists, and unavailability of assistive technology [7]. Furthermore, in many cultures, social stigma of having a child with a disability prevents parents from visiting health care [7]. The World Health Organisation, WHO, estimated that 80 percent of the world’s population with disability live in resource-poor settings [8]. Gladstone concluded in a review of childhood CP in resource-poor settings, that the prevalence of CP was difficult to obtain and that there is a great need of classifications [8].

Recently, the World Health Assembly adopted a resolution endorsing the WHO global disability action plan 2014–2021: “Better health for all people with disability”. The three objectives of the action plan are; 1. “To remove barriers and improve access to health services and programmes”, 2. “To strengthen and extend rehabilitation, habilitation, assistive technology, assistance and support services, and community-based rehabilitation”, 3. “To strengthen collection of relevant and internationally comparable data on disability and support research on disability and related services” [9].

One of the prerequisites for approaching these goals is a common language, i.e. classification systems that describe characteristics of the group in question. However not until the late 1990s was the first reliable classification system available, the Gross Motor Function Classification System (GMFCS), [10]. Prior to this, descriptions (e.g. mild, moderate, and severe) and scales for mobility and gross motor function were used to describe the child’s level of disability [11, 12]. One of these scales, with a construct closely related to the GMFCS, is the Wilson mobility scale, a nine-level ordinal scale, indicating the child’s present performance of mobility [12–14]. The description of each level is short and the scale has been considered easy to use. Distinctions are made between walking in all surroundings and in secluded (sheltered) surroundings, and between walking with and without walking aids. Furthermore, the last three levels in descending order in the Wilson mobility scale, includes one level for reciprocal (alternating arm and leg movements) crawling, one level for any other form of locomotion (except crawling and walking), and the description of level nine is: Sitting with support and no mobility (Table 1).

**Table 1:** Wilson mobility scale presented in Venezuelan Spanish and in English. Secluded (sheltered) surroundings, key-walker (posterior walker), reciprocal (alternately moving right arm and left leg and vice versa) crawling

Nivel de función según Wilson	
1.	Marcha funcional sin ayuda en todo tipo de ambientes
2.	Marcha funcional sin ayuda en ambientes protegidos
3.	Marcha funcional con muletas en todo tipo de ambientes
4.	Marcha con muletas en ambientes protegidos
5.	Marcha functional con andador en todo tipo de ambientes
6.	Marcha con andador en ambientes protegidos
7.	Gateo recíproco
8.	Algun tipo de locomoción, describala
9.	Se sienta con apoyo del respaldar sin ninguna locomoción
Wilson mobility scale	
1.	Functional walking without aid in all surroundings
2.	Functional walking without aid in secluded surroundings
3.	Functional walking with crutches in all surroundings
4.	Walking with crutches in secluded surroundings
5.	Functional walking with key-walker in all surroundings
6.	Walking with key-walker in secluded surroundings
7.	Reciprocal crawling with arms and legs
8.	Any other form of locomotion
9.	Sitting with support and no mobility

In 1997 the Gross Motor Function Classification System (GMFCS) was developed to provide a standardized system for classifying the child’s present gross motor ability [10]. It was the first classification system for children with CP to be validated and tested for reliability and stability [15, 16]. Later, the GMFCS was expanded and revised to also include individuals within the age-span from 12–18 years, the GMFCS–E&R [17] (Table 2). The focus of the classification is the child’s self-initiated movement, with emphasis on sitting, walking, and wheeled mobility. Everyday activity is stressed; what the child usually does do (performance), rather than what the child optimally can do (capacity) [18]. The classification includes five levels, and within each level there are five age-spans (<2 year, 2–4, 4–6, 6–12, and 12-18 years). Distinctions between levels reflect the child’s present abilities and the need for assistive equipment. The distinctions enable clinicians, parents, and researcher to consider whether an intervention or prognosis is relevant for a specific child, which is of importance since there is a large variability within the group of children with CP [17, 19]. The use of GMFCS-E&R comprises additional advantages. It can facilitate discussions concerning realistic goal-setting and need of assistive devices. Furthermore, a classification system enables comparisons between different interventions and across regions [19]. As of spring 2015, the GMFCS-E&R was translated into 24 languages [19]. However, from a global perspective the classification was only rarely used in resource-poor settings in large parts of the world [8, 20]. South America, Africa and Asia are large, highly populated continents where only a minor part of the countries are aware of and use GMFCS-E&R to describe gross motor function in children with CP [21]. Professionals as well as advocacy groups from these continents have stressed the fact that the classification are rarely used and that there exist many barriers which often relate to circumstances in the local context (lack of knowledge, economic, religious, social stigma, etc.) The African Child Policy Forum reports an absence of reliable data of children with disabilities and conclude that the reason originates in part from a lack of standardized definitions of disability but also from the absence of distinction between the degrees of severity of impairment [21]. Thus it is desirable to develop a low-cost model for the introduction of the GMFCS in the actual context where it will be used together with local professionals.

**Table 2:** Gross Motor Function Classification System- Extended and Revised (GMFCS-E&R) in the age band between 6^th^ and 12^th^ birthday, presented in Spanish and in English

GMFCS-E&R	Generalidades De Cada Nivel	(Spanish)
Nivel I - Camina sin restricciones		
Nivel II - Camina con limitaciones		
Nivel III - Camina utilizando un dispositivo manual auxiliar de la marcha		
Nivel IV - Auto-movilidad limitada, es posible que utilice movilidad motorizada		
Nivel V - Transportado en silla de ruedas		
GMFCS-E&R	General Headings For Each Level	(English)
Level I - Walks without Limitations;		
Level II - Walks with Limitations		
Level III - Walks Using a Hand-Held Mobility Device		
Level IV - Self-Mobility with Limitations, May Use Powered Mobility		
Level V - Transported in a Manual Wheelchair		

The specific aims of this study were to introduce the Gross Motor Classification System Expanded and Revised (GMFCS-E&R) version in a setting with no previous knowledge of this classification system and to examine the validity and reliability. An additional aim was to examine a model, within the actual environment at the targeted facilities that included a layman facilitator speaking the local language as well as the language of the instructors.

### Brief statement

The model to introduce the GMFCS-E&R version in a setting with no previous knowledge consisted of a short period of education, tutoring, self-study, and practice for one Venezuelan paediatrician and one layman facilitator speaking both the language of the instructors and the target country. The results indicated that the model was efficient and gave rise to very good inter-rater reliability, and concurrent validity was confirmed.

## Methods

### Design

The study was a cross-sectional reliability study and a collaboration between Centro de Rehabilitación Infantil "Mundo de Sonrisas" Puerto Ordaz, Venezuela and The Department of Women's and Children's Health, Karolinska Institutet, Stockholm, Sweden.

## Setting

The Child Rehabilitation Centre is one out of two secondary rehabilitation facilities available in Venezuela, serving part of the 1.6 million inhabitants and 285.000 km^2^ size Bolivar State (for reference- slightly larger than the United Kingdom). About 1300 children are regularly seen at the Centre and 271 of these have a diagnosis of CP.

## Participants

Children were invited to participate during October-November 2013. The inclusion criteria were; children 3–18 years of age, a diagnosis of cerebral palsy including all subtypes and distributions. The exclusion criteria were; no possibility of contacting the family such as absence of mobile phone or families living very remote or inaccessible, i.e. in the jungle or out of reach for public transportation.

The inter-rater reliability was assessed between; the paediatrician in Venezuela and the student visiting Venezuela, between the paediatrician in Venezuela and the physiotherapist (PT) in Sweden and between the student visiting Venezuela and the PT in Sweden.

Ethical permission was obtained through Fundación Social Bolívar, Gobernación del Estado Bolívar. The families were given oral information of the study and oral consent from the parents was obtained, including permission for the child to be filmed.

## Instruments: classification, scale and questionnaire

### The Spanish version of GMFCS-E&R, Clasificación de la Función Motora Gruesa

Extendida y Revisada, downloaded from the CanChild official website (http://www.canchild.ca/en), was used without further adaptations to Venezuelan Spanish (Table 2).

The Wilson mobility scale was used with adaptations to Venezuelan Spanish. The Wilson mobility scale is a nine level, ordinal scale ranging; from 1: independent walking in all surroundings, to 9: no mobility [12–14] (Table 1). The Wilson mobility scale was chosen to examine the concurrent validity of the GMFCS-E&R since it 1) besides the description of walking with and without assistive devices 2) also includes alternative methods of mobility, and 3) in addition a description of the need of support in sitting.

A structured questionnaire was constructed to facilitate the discussion with the parent/caregiver. The aims were to collect information concerning the child’s performance and need of assistance in various environments and thereby obtain relevant information in order to make appropriate classifications and facilitate the scoring. In addition, the information from the questionnaire was a prerequisite for the assessment in Sweden. The structured questionnaire included seven questions that comprised aspects of the child’s best capacity and everyday performance of mobility within and outside the home, gross motor ability such as; sitting, moving around, walking distance, alternative method of mobility and the use of assistive devices or need of caregiver assistance.

## Procedure

The study consisted of four stages; 1. The paediatrician and the student were educated. 2. The Wilson mobility scale and the structured questionnaire were translated. 3. The evaluators in Venezuela independently assessed and documented the children. 4. The PT in Sweden independently assessed the collected data.

### The initial stage - education and supervising

The Swedish paediatric neurologist sent the Spanish version of GMFCS-E&R to the paediatrician in Venezuela and discussed the use of it. The paediatrician studied the user instructions and practiced for some weeks at regular outpatient visits, in order to learn and familiarize with the classification.

A Swedish high school graduate (referred to as the student), fluent in Spanish and representing a layman, was educated during a total of five hours before the visit to Venezuela. The education contained the user instructions and principles of the GMFCS-E&R, taught by an experienced physiotherapist, and by observing and scoring movies of children in all GMFCS-E&R levels. The Swedish student was instructed in the principles of the Wilson mobility scale and in the questions within the structured questionnaire that was going to be used.

#### The second stage - translation

To additionally explore and confirm the present mobility of the child, the Wilson mobility scale was used. The Wilson mobility scale was translated and back translated from English to Venezuelan Spanish. All translations were discussed and revised to ensure consistency before being finalized. The last step involved a back translation from Venezuelan Spanish to English by an independent paediatrician [22]. The group involved in the translation process included; two paediatricians from Venezuela, one paediatrician from Argentina, one neurologist from Peru and one paediatric neurologist from Sweden. The group had excellent English, Spanish and Swedish language abilities.

To facilitate the discussion with the families and children and further explore and confirm the present performance of the child, a structured questionnaire was constructed. The structured questionnaire was translated from Swedish to Venezuelan Spanish by a Venezuelan paediatrician fluent in Swedish and well familiar with Venezuelan way of expression.

#### The third stage - child assessment in Venezuela

The children’s families were contacted by telephone and invited to take part in the study by a physiotherapist in Venezuela. The families that consented to participate visited the centre "Mundo de Sonrisas" in Puerto Ordaz. Each child was independently assessed by the paediatrician (YA) and the student (MT), who assigned a GMFCS-E&R level and a Wilson mobility scale score. In addition, the paediatrician (YA) recorded the diagnosis and information about pregnancy, gestational age, birth and seizure disorder. Furthermore the student (MT) filmed each child and interviewed the parent/caregiver utilizing the structured questionnaire.

#### The fourth stage - assessment and classification in Sweden

In Sweden the pediatric neurologist (KT) reviewed all films to confirm the diagnosis and subtype of CP. Then the physiotherapist (KL) with long experience of GMFCS-E&R and the Wilson mobility scale independently classified the children (denoted the level in GMFCS-E&R and the Wilson mobility scale score). This was accomplished through merging the data from the structured questionnaire with observations of the films, without access to previous classification and scoring from the assessments performed in Venezuela.

## Statistics

Descriptive parametric and non-parametric statistics were used and presented as mean, standard deviation, median, range and 25^th^-75^th^ percentiles. Cohen’s weighted kappa was used to calculate the agreement of the inter-rater reliability. Since both the GMFCS-E&R and the Wilson mobility scale represent ordinal data, non-parametric statistics were required and the results were presented with weighted Kappa (Kw) [23, 24]. The interpretation of the strength of agreement was completed according to Landis and Koch in which a Kappa value of; less than 20 is poor, 0.21-0.40 is fair, 0.41-0.60 is moderate, 0.61-0.80 is good and 0.81-1.0 is very good [25]. Spearman’s correlation coefficient was utilized to calculate correlations between the GMFCS-E&R and the Wilson mobility scale to investigate the concurrent validity. Correlations were considered significant if they reach both a p-value <0.05 and r_s_ >0.47. The interpretation according to Cohen was used [26]. Statistical analysis was performed using the Statistical Package for Social Sciences (SPSS) version 22.

## Results

During the months of enrolment, 120 children were invited to take part in the study and 91 families agreed to participate. Eighty-eight children fulfilled the inclusion criteria and were included. Within this group of children 56 were boys and 32 were girls with a mean age of 10 years (SD: 4, range; 3-18years). The mean gestational age at birth was 36 weeks (SD: 4 weeks). Thirty-one children (35 %) were born prematurely with a mean gestational age of 32 weeks (SD: 3.5 weeks, range: 26-37weeks). Intrauterine infection was described in 36 children (41 %). In 14 children (16 %) the CP had a postnatal aetiology, infection in 13 children (15 %) and traumatic brain injury in one child. Seizure disorder was present in 30 children out of 85 (34 %) and the information was not available for three children.

All types of CP were represented and 60 children (68 %) had a bilateral spastic CP, 15 children (17 %) a unilateral CP, seven children a dyskinetic CP (8) and six children (7 %) had an ataxic CP. All five levels within the GMFCS-E&R were represented whereof; 19 (22 %) children were classified in GMFCS-E&R I, 23 (26 %) in GMFCS-E&R II, 14 (16 %) in GMFCS-E&R III, 15 (17 %) in GMFCS-E&R IV and 17 (19 %) of the children were classified in GMFCS-E&R V (Table 3). The Wilson mobility scale indicated the children’s mobility. The median value for the total group was 5 (25^th^-75^th^ percentile: 1–8, range: 1–9). Thirty-two (36 %) children received a score of 1, implying independent walking in all surroundings. A score of 9 was present in 18 (21 %) children, implying sitting with support and no mobility (Table 3).

**Table 3:** Descriptive statistics presented as mean, standard deviations (SD), median, number (no) and range, (*n* = 88)

Descriptive statistics	
Participants (no)	88
Gender (no)	
Male	54
Female	34
Age	
Mean (SD)	10 (4)
range	3-18
Diagnosis (no)	
Spastic bilateral	60
Spastic unilateral	15
Dyskinesia	7
Ataxic	6
GMFCS-E&R level (no (%))	
GMFCS I	19 (22)
GMFCS II	23 (26)
GMFCS III	14 (16)
GMFCS IV	15 (17)
GMFCS V	17 (19)
Gestational age at birth (weeks)	
Mean (SD)	36 (4)
Intrauterine infection (no)	
Yes	36
No	51
Information unavailable	1
Postnatal cause (no)	
None	71
Infection	13
Traumatic brain injury	1
Information unavailable	3
Seizure, current (no)	
Yes	30
No	55
Information unavailable	3
Wilson mobility scale (no)	
1	32
2	10
5	6
6	3
7	2
8	17
9	18

The inter-rater reliability of GMFCS-E&R was calculated between pairs of examiners. Agreement; between the paediatrician (YA) and the PT (KL) was Kw = 0.85 (95 % CI: 0.75-0.88), between the student (MT) and PT (KL) was Kw = 0.91 (95 % CI: 0.86-0.95) and between the paediatrician (YA) and student (MT) was Kw = 0.85 (95 % CI: 0.79-0.90). The inter-rater reliability of the Wilson mobility scale was also calculated between pairs of examiners. Agreement; between the paediatrician (YA) and the PT (KL) was Kw = 0.86 (95 % CI: 0.80-0.93), between the student (MT) and PT (KL) was Kw = 0.96 (95 % CI: 0.93-0.99) and between the paediatrician (YA) and student (MT) was Kw = 0.94 (95 % CI: 0.90-0.97) (Table 4). The disagreements were mostly caused by the non-availability of walking aids.

**Table 4:** The The inter-rater reliability of the GMFCS-E&R and the Wilson mobility scale was calculated between pairs of examiners (Paediatrician, Physiotherapist (PT) and the Layman/Student). The weighted Kappa coefficient (Kw) and the 95 % Confidence interval (95 % CI) were presented. Eighty-eight children with cerebral palsy were examined

Inter-rater reliability Kw (95 % CI)	Paediatrician - PT	PT- student	Paediatrician -student
GMFCS-E&R	0.85 (0.75-0.88)	0.91 (0.86-0.95)	0.85 (0.79-0.90)
Wilson mobility scale	0.86 (0.80-0.93)	0.96 (0.93-0.99)	0.94 (0.90-0.97)

Concurrent validity was present, the correlation between GMFCS-E&R and the Wilson mobility scale was high for all examiners; the paediatrician (YA) r_s_ =0.94 (*p* < 0.001), the student (MT) r_s_ =0.95 (*p* < 0.001), and the PT (KL) r_s_ =0.95 (*p* < 0.001).

## Discussion

The overall aim of this study was to examine a model to introduce GMFCS-E&R in a setting with no previous knowledge of the classification and furthermore to determine the validity and the reliability. The main findings revealed that the model with education, tutoring, self-study, and practice, was efficient and gave rise to very good reliability and concurrent validity between the GMFCS-E&R and the Wilson mobility scale.

In the WHO global disability action plan, the overall aim is to offer better health for all people with disability. A central tenet in this work is to provide evidence based interventions. Since the CP diagnosis is an umbrella term covering a heterogeneous group of children, interventions can vary greatly [27, 28].The use of classifications is therefore essential, which has been proposed in the recent definition of CP (21). The major intention of a classification is to offer meaningful distinctions among various stages or expressions in a health status, and thereby provide possibilities for prognosis and relevant treatment options [19]. In addition, the global disability action plan aim to “strengthen collection of relevant and internationally comparable data”, and classifications are one among other prerequisites to reach advancement towards that goal. In a recent study exploring motor severity in children with CP, in a high- (Australia) and low-resource (Bangladesh) country, the authors concluded that there was limited application of a classification system such as GMFCS-E&R in the low-resource country [20]. They suggested training of health professionals in such classification systems, which would provide possibilities for prognosis and relevant interventions [20].

The present study was performed as a collaboration between Centro de Rehabilitación Infantil "Mundo de Sonrisas" Puerto Ordaz, Venezuela and the Department of Women's and Children's Health, Karolinska Institutet, Stockholm, Sweden. The age span, 3–18 years, was selected in order to facilitate the reliability testing of GMFCS-E&R. Children below two years of age were excluded since the reliability in that age-span has been questioned [29]. Within the group of children participating in the study, the representation of type and distribution of CP contrasted, to some extent, to what has been reported from North America, Canada and the European (SCPE) database [30, 31]. In the present study, there were a higher number of children with a bilateral spastic CP and fewer children with a unilateral spastic CP. Perhaps the discrepancy could be explained by the families’ perceived need of health care, implying that families with children having unilateral spastic CP more rarely visited the hospital. Children classified within all five GMFCS-E&R levels were represented (Table 2). In comparison to a population study from Sweden, the representation of various levels demonstrated a discrepancy where the most significant difference was the lower number of children classified in level I in the present study [32]. However the representation corresponded to a convenient sample of children participating in a reliability study in Brazil [33]. A lower number of children in GMFCS-E&R level I-II were also observed in Bangladesh, reported in the study comparing children with CP in Bangladesh and Australia [20]. Perhaps the lower number of children in GMFCS-E&R level I to some extent also could be explained by less frequent visits to the hospital since the perceived need of health care was less for children in GMFCS-E&R level I.

Among the participants, 36 % were born prematurely, which is consistent with the results from a population based study in western Sweden reported by Himmelmann and Uvebrant [34] but somewhat fewer than reported from the SCPE database [31]. Presence of a seizure disorder was reported in 34 % and corresponds to what has been reported from other parts of the world [35, 36].

The results with very good agreement between all examiners, both concerning the GMFCS-E&R and also the Wilson mobility scale correspond well with another recent GMFCS-E&R reliability study performed in Brazil [33]. Silva and collaborators made their inter-rater reliability testing by analysing the agreement of the classifications made from video recordings, a procedure to some extent similar to the one performed (in Sweden) in the present study. However, in the present Venezuelan-Swedish study the addition of the structured questionnaire proved to contribute with valuable and essential information for the classification procedure. When (GMFCS-E&R) classifying is made only from a video recording, there is a risk that the filmed sequence represents the child’s “best-possible ability” and that limited information is provided on how the child usually performs in various settings. This risk is in part illustrated in the Brazilian study where they also studied the reliability between professionals and parents, which demonstrated a lower agreement than the reliability between professionals. The authors concluded that the parents classified their children in levels with more limitations since they knew the performance in different environments, while the professionals observed children in only a single situation [33].

In a Korean version of the GMFCS, Ko and co-workers also determined the reliability and concurrent validity [37]. They used the Pediatric Evaluation of Disability Inventory (PEDI) as a comparison to the GMFCS. The result demonstrated moderate to good concurrent validity. The highest correlation was observed between GMFCS and the PEDI functional skills scales of mobility. Furthermore, the inter-rater reliability was very good and the level of experience among the raters did not affect the result, findings which our results confirmed [37].

The high reliability in the present study could possibly be explained by the structured way the assessors in Venezuela operated when denoted the level within GMFCS-E&R and the score in the Wilson mobility scale. They strictly followed the user instructions, since they were novices in using the classification and the scale. The combination of using the GMFCS-E&R, the Wilson mobility scale and the structured questionnaire were considered as a trustworthy method to achieve the actual picture of each child’s performance. The structured questionnaire included questions both on the child’s best capacity as well as the daily performance, which facilitated the GMFCS-E&R classification (performance). However, the Swedish student noted that some parents never tested the limit of their child’s abilities. Possibly as a result of cultural differences in parental interacting behaviour; some parents acted in concordance with a tradition where parents are obliged to help their children with disabilities at all times , implying that the children receive more assistance rather than being raised or trained towards independence.

One could perhaps question why the Wilson mobility scale was used to test the concurrent validity, since there are other scales that have been more frequently used in children with CP. The Gillette Functional Walking Scale for example, is often used and is a ten level ordinal scale [11]. The scale describes walking in an ascending order, from not being able to take any step, to be able to walk, run and climb on uneven terrain without difficulty. Since the scale has previously demonstrated positive results, when tested for reliability and validity, it would have been possible and correct to use it [11]. However, the Gillette Functional Walking Scale only describes a broad range of walking abilities but no alternatives to walking are included, thus, the Wilson mobility scale was preferred since a broader perspective on the mobility was required to cover GMFCS-E&R.

Initially, the paediatrician and student felt that the Wilson mobility scale was easier to use due to the short explanations for the different levels. However, subsequently, they believed that the more comprehensive descriptions (e.g. where sitting is also addressed) in GMFCS-E&R provided additional valuable aspects of the child’s functioning. Thus, during the time of the study and since mobility devices was frequently missing, the GMFCS-E&R turned out to be easier to match with the child’s present performance. Nevertheless, the correlations between the level within GMFCS-E&R and the Wilson mobility score were all very high, implying concurrent validity due to a similar construct.

The disagreements between the examiners were mostly caused by the absence of assistive devices, implying confusions, since the present ability of the child did not perfectly match any description. The non-availability of walking aids was due to several factors; a general shortage of assistive devices, the economic situation of the families as well as the fact that some families lived in the Amazonas or rural areas where the roads where not adapted for technical aids. The low degree of access to assistive devices were also described for children in Bangladesh, which implied that, for example, children in GMFCS-E&R III to a lesser extent were able to walk, since they did not have access to walkers [20]. The worldwide situation is that only an estimated 10–15 percent of individuals with disabilities have actual access to assistive devices/technologies (http://www.who.int/disabilities/technology/en/).

This study had some limitations; the most important was probably the fact that the PT in Sweden classified and scored children based on information from the structured questionnaires and the films, without being able to discuss with the child and family. Additionally, the confirmation of the diagnosis and classification by the paediatric neurologist in Sweden, were based on reviewing the films and written documentation.

## Conclusions

In a setting with no previous knowledge of GMFCS-E&R the combination of using the GMFCS-E&R, the Wilson mobility scale and the structured questionnaire were considered as an efficient way of introducing GMFCS-E&R. The Wilson mobility scale confirmed the assignments of GMFCS-E&R levels, since associations between GMFCS-E&R and the Wilson mobility scale were high. The model with a short period of structured learning, tutoring and practice gave rise to high reliability. The disagreements were mostly caused by the non-availability of walking aids. In this study, none of the trained and skilled professionals in Sweden knew the language in the local setting. The possibility to use the same model, with a layman facilitator speaking both the language of the instructors and the target country, opens up opportunities for future knowledge dissemination to other countries. Thus, the overall objectives of the study were met but this can only be considered as the first small step on a longer path. The next step, implementation of GMFCS-E&R in the clinical practice, requires further extensive work.

## Abbreviations

CP: Cerebral palsy
GMFCS-E&R: Gross Motor Function Classification System-Expanded and Revised
